# Anterolateral thigh free flap in reconstruction of lateral skull base defects after oncological resection

**DOI:** 10.1007/s00405-019-05627-x

**Published:** 2019-09-12

**Authors:** Piotr Trojanowski, Marcin Szymański, Agnieszka Trojanowska, Adrian Andrzejczak, Dariusz Szczepanek, Janusz Klatka

**Affiliations:** 1 Department of Otolaryngology and Laryngological Oncology, Medical University of Lublin, Jaczewskiego 8, 20 954 Lublin, Poland; 2 I Department of Medical Radiology, Medical University of Lublin, Lublin, Poland; 3 Department of Neurosurgery and Paediatric Neurosurgery, Medical University of Lublin, Lublin, Poland

**Keywords:** Anterolateral flap, Lateral skull base reconstruction, Malignant tumour, Temporal bone

## Abstract

**Purpose:**

Evaluation of the utility of the free anterolateral thigh flap reconstruction of the defects resulting from radical temporal bone resection in the management of lateral skull base malignancies in a single institution.

**Methods:**

An analysis of 17 en bloc subtotal petrosectomies for removal of malignant tumours was performed. There were 12 squamous cell carcinomas, 4 basal cell carcinomas and 1 adenoid cystic carcinoma. The tumours were staged with the University of Pittsburgh TNM system. In all patients, the lateral temporal bone with the preservation of the petrous apex and carotid artery was performed. All patients had parotid gland resection. The post-resection defect was reconstructed with an ALT free flap.

**Results:**

Tumour radical resection and defect reconstruction with an ALT flap was achieved in all patients without intraoperative complications. The transplants were harvested as fasciocutaneous flaps, 11 perfused by musculocutaneous and 6 by septocutaneous perforators. The ALT flaps had a mean pedicle length of 8 cm (6–12 cm), and the flap size ranged between 6 × 15 cm and 15 × 30 cm. The flaps were supplied by nine facial, five occipital and three maxillary arteries. Recipient-site veins included eight internal jugular, seven facial, one retromandibular and one external jugular vein. All arterial pedicles were anastomosed in an end-to-end manner. The veins were anastomosed with interrupted sutures and in 11 cases with Synovis-Coupler® devices. All the flap transfers were performed successfully. Three patients experienced postoperative complications.

**Conclusions:**

The ALT flap proved to be effective for covering large temporal skull base defects resulting from the radical removal of temporal bone malignancies. The functional and cosmetic results were acceptable with a low complication rate.

## Introduction

Lateral skull base malignancies present a significant clinical challenge for the head and neck surgeons. Those malignancies include very rare primary tumours of the temporal bone and about ten times more frequent cancers of the parotid gland and skin. Malignant tumours of the external auditory canal, auricular and periauricular skin and parotid gland may spread by direct invasion into the temporal bone and its neighbouring structures [1–7].

Radical treatment of those malignancies usually requires en bloc resection of all the involved structures. In case of temporal bone invasion, different options of surgical procedures are utilised: lateral temporal bone resection, subtotal petrosectomy and total petrosectomy. In lateral temporal bone resection, the bony and cartilaginous external auditory canal, tympanic membrane, malleus and incus are removed, with the preservation of the vertical segment of the facial nerve. Subtotal petrosectomy involves removal of the lateral temporal bone, saving the petrous apex and preservation of the carotid artery. In total petrosectomy, the entire temporal bone is removed, sometimes with carotid artery sacrifice [1, 2, 8–10].

Complex anatomy of the area contributes to the difficulty of surgery and creates a major reconstructive challenge. Development of the free tissue transfers in reconstructive surgery offered new horizons in surgical treatment of lateral skull base malignancies. Availability of adequate reconstruction methods allows more radical approaches, with regard to the resection margins. Good reconstruction of the surgical defects improves the results of treatment in terms of mortality and postoperative morbidity. Spacial complexity of the defects, a large amount of bulk necessary to obliterate dead space, lack of elasticity of the proximal and distal neighbouring bone and soft tissue requires refined reconstruction techniques. Reconstruction should preserve residual functions, reduce morbidity and does not impair mobility of the structures around the resected area with acceptable cosmetic effect [11–18].

An adequate transplant needs to provide pliable tissue, adequate in volume and able to provide robust and vascularised coverage of major vascular and nervous structures and even the exposed brain. In the direct neighbourhood of the defect, an adequate amount of tissue is not present. Therefore, the use of transplants from distant sites became a valid solution. In patients previously operated and/or irradiated, reconstructive procedures are even more demanding. In those planned for postoperative radiation therapy, reconstruction should be solid enough to withstand irradiation.

A variety of free flaps are used to cover the post-resection defects in the lateral skull base. An anterolateral thigh flap (ALT) is often used in the head and neck region reconstruction, but in the repair of the lateral skull base defects, the experience is limited. Particularly, the evaluation of the recipient and the donor-site complications are unequivocal in the recent literature [14–16].

## Aim

Evaluation of the utility of the free anterolateral thigh flap reconstruction of the defects resulting from radical temporal bone resection in the management of lateral skull base malignancies in a single institution.

## Material and methods

Between 2014 and 2018, en bloc subtotal petrosectomy in the treatment of 17 malignant tumours was performed in 15 male and 2 female patients, ranging between the ages of 47 and 87 years. Primarily, the tumour originated in the external auditory canal in one case, auricle in three, periauricular skin in seven and in six patients in the parotid gland. The pathological entities included 12 squamous cell carcinomas, 4 basal cell carcinomas and 1 adenoid cystic carcinoma. In three cases, it was a recurrence after periauricular skin squamous cell carcinoma resection and irradiation.

The tumours were evaluated with the University of Pittsburgh TNM staging system [4]. In 13 patients, it was stage T4 and the remaining 4, stage T3. The patients had intact facial nerve function prior to surgery in three cases, while the remainder presented clinical facial nerve dysfunction from a presumed tumour infiltration. The patient’s physiological status predicting operative risk was assessed using American Society of Anesthesiologists Classification (ASA Class) [19]. There were eight patients ASA IV, 5 ASA III and 4 ASA II.

In all patients, en bloc resection—subtotal petrosectomy involving the removal of the lateral temporal bone with the preservation of the petrous apex and carotid artery was performed. All patients had parotid gland resection: 14 of them had total parotidectomy with facial nerve sacrifice, 3 of them had superficial parotidectomy with facial nerve preservation. In three cases, the excised facial nerve was reconstructed intraoperatively. In one case, with stump aproximation and primary neurorrhaphy and in two cases, with a nerve graft. In five patients, partial mandibulectomy with temporomandibular joint and in four, a zygomatic arch and infratemporal fossa resections were performed. Neck dissection was executed in all patients. In nine of them, it was elective, and in eight, it was therapeutic (Table 1).

**Table 1:** Surgical treatment–tumour resection in 17 patients with lateral skull base malignancies

	Number
Type of surgery	
Lateral skull base resection	0
Subtotal petrosectomy	17
Total petrosectomy	0
Parotidectomy	
Superficial	3
Total	14
Resection of adjacent structures	
Intentional excision of FN	14
Dura resection	2
Partial mandibulectomy and temporo-mandibular joint	5
Zygomatic arch and infratemporal fossa	4
Neck dissection	
Elective	9
Curative	8

In all the patients, the post-resection defect was reconstructed with a free ALT flap. Table 2 presents individual patients’ characteristics.

**Table 2:** Patient’s characteristics

Patient	1	2	3	4	5	6	7	8	9	10	11	12	13	14	15	16	17
Age	72	63	85	64	83	87	75	87	84	86	65	82	76	47	82	73	62
Gender	m	m	m	m	m	m	m	m	f	m	f	m	m	m	m	m	m
Diagnosis																	
Tumour origin																	
Parotid	x				x						x	x	x				x
Periauricular skin		x	x						x	x				x	x	x	
Auricle				x		x	x										
EAC								x									
Pathology	scc	scc	bcc	bcc	scc	scc	scc	bcc	scc	scc	scc	scc	adc	bcc	scc	scc	scc
Staging, T	4	3	4	4	4	4	4	4	3	4	4	4	4	3	3	4	4
Comorbidities	l, h, ci,s	a	h		h, mi, d, ci	ci	mi, h, st, ca	cp, d	cb, h	h		a, ci	ci, h, d, ca	d	h, ci	cl, hy	hy
Extension of resection																	
Subtotal petrosectomy	x	x	x	x	x	x	x	x	x	x	x	x	x	x	x	x	x
Parotidectomy	total	total	total	total	total	total	total	total	superf	total	total	total	total	superf	superf	total	total
Facial nerve	reS	reC	reS	reC	reS	reS	reS	reS	Pre	reS	reC	reS	reS	Pre	Pre	reS	reS
TMJ					x		x	x					x				x
dura			x				x										
Zygomatic a. infratemporal f	x		x							x						x	
ALT																	
Fasciocutaneous flap	x	x	x	x	x	x	x	x	x	x	x	x	x	x	x	x	x
Perforators																	
Septal									x	x	x	x	x			x	
Muscular	x	x	x	x	x	x	x	x						x	x		x
Flap dimensions cm	16 × 8	15 × 10	16 × 7	17 × 9	17 × 8	21 × 10	15 × 11	15 × 7	16 × 8	16 × 7	15 × 6	23 × 10	22 × 10	21 × 11	18 × 9	17 × 11	30 × 15
Pedicle																	
Length, cm	7	6	8	7	12	8	11	8	9	7	9	6	8	7	6	8	10
Type	trans	desc	desc	desc	desc	desc	desc	desc	desc	trans	desc	desc	desc	desc	desc	trans	desc
Anastomosis																	
Artery	max	occ	occ	fac	fac	fac	fac	max	fac	fac	fac	max	fac	occ	fac	occ	occ
Vein	jag	jag	fac	fac	jag	retr m	jag	fac	ext	jag	jag	jag	fac	jag	fac	fac	fac
Complications					necro	haem	mi haem						mi				

*EAC* external auditory canal, *scc* squamous cell carcinoma, *bcc* basal cell carcinoma, *ac* adenoid cystic carcinoma, superf superficial parotidectomy, *reS* facial nerve resection, *reC* facial nerve reconstruction, *Pre* fascial nerve preservation, *TMJ* temporo-mandibular joint, *trans* transvers, *desc* descending, *max* maxillary, *occ* occipital, *fac* fascial, *jag* jugular, *retr* m retromandibular, *ext* external jugular, *necro* flap necrosis, *haem* haematoma, *mi* myocardial infarction. Comorbidities: h hypertension, *ci* c, *d* diabetes, *l* leukemia, *cap* prostate ca, *cb* breast ca, *cl* lung ca, *hy* hypothyroidism, *st* stroke, *s* sarcoidosis, *mi* myocardial infarction

## Results

**Fig. 1:**
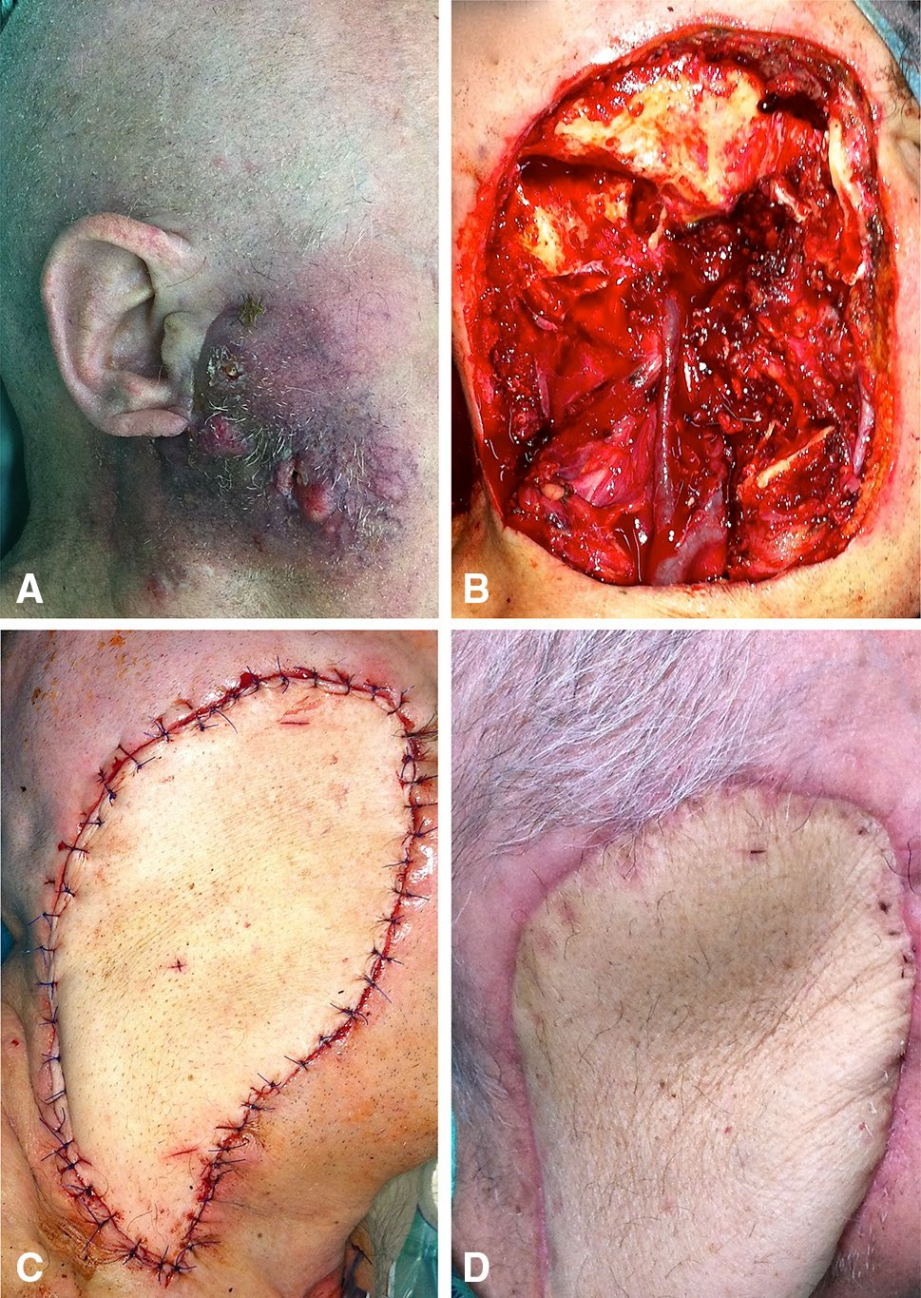
**a** 72-year-old man with a recurrence of squamous cell cancer of the right parotid gland. **b** Defect following resection of right lateral temporal bone, right parotid gland with facial nerve, external auditory canal, infratemporal fossa, and mandibular segment of the temporomandibular joint. **c** Intraoperative right profile view of fascio-cutaneous ALT flap reconstruction of right lateral temporal bone defect. **d** 18 month postoperative view of the ALT flap reconstruction

**Fig. 2:**
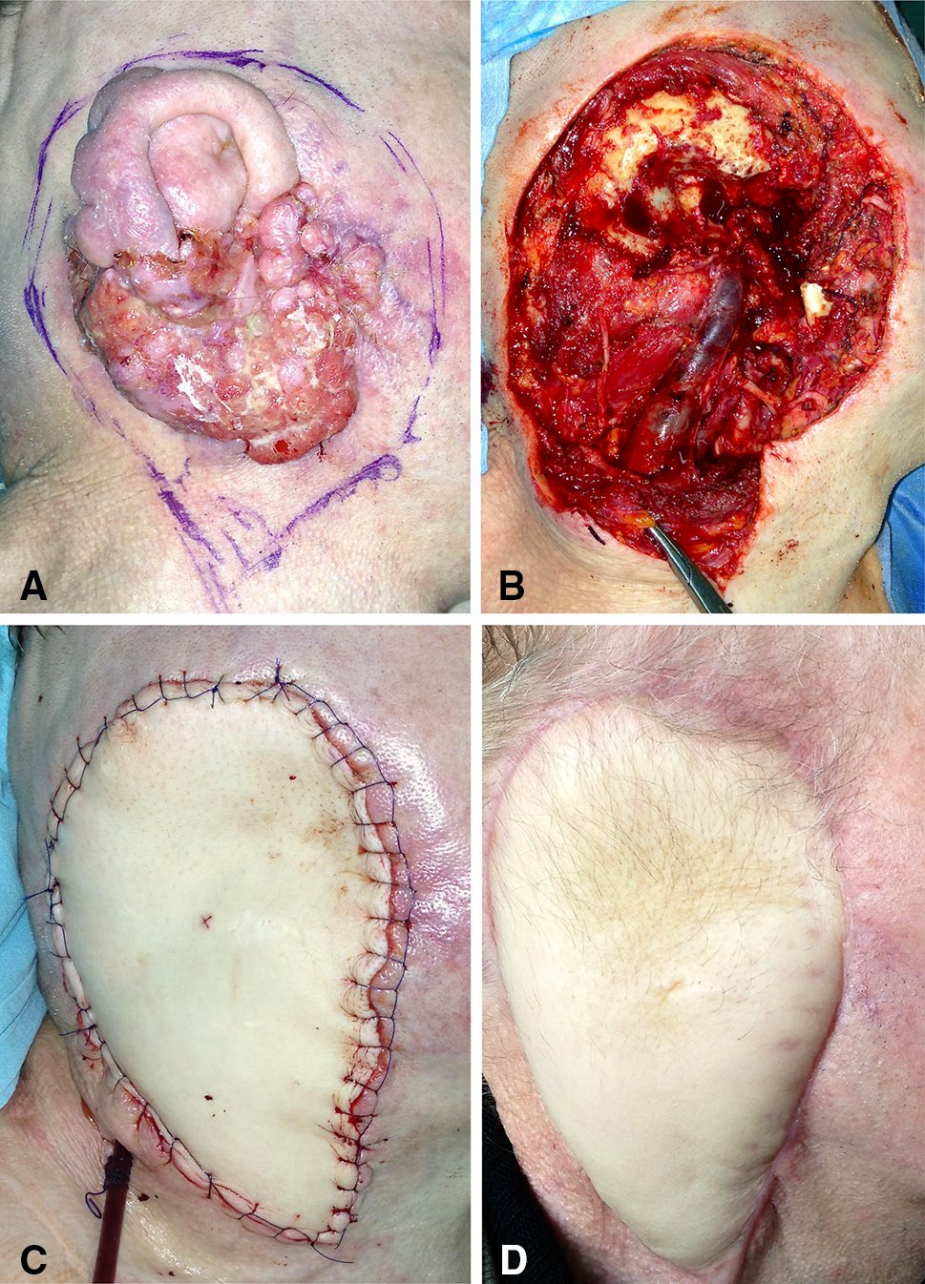
**a** 67-year-old man with a squamous cell carcinoma of right auricle. Planned resection margins marked. **b** Defect following resection of right lateral temporal bone, external auditory canal, auricle and right parotid gland. **c** Intraoperative right profile view of fascio-cutaneous ALT flap reconstruction of right lateral temporal bone defect. **d** Lateral view of right temporal bone reconstruction at 12 months postoperative follow up

In all cases, resection of the tumour and reconstructive surgery with ALT flap was carried out without any intraoperative complications (Figs. 1, 2). The ALT flaps were harvested as a fasciocutaneous flaps. The perforators, supplying the skin island, arose from the descending branch of the lateral circumflex femoral artery (LCFA) in 14 patients. The perforator arising from the transverse branch of the LCFA was encountered in three cases. In one case, no sufficient perforator could be found, and the flap had to be harvested from contralateral thigh and supplied by a descending LCFA branch.

Out of 17 flaps, 11 were perfused by musculocutaneous perforators, while 6 were perfused by septocutaneous perforators. The ALT flaps had a mean pedicle length of 8 cm with a range between 6 and 12 cm. The artery diameter was around 2 mm. There were always two concomitant veins encountered, with the diameter varying from 1 to 4 mm. The overall flap size ranged between 6 × 15 cm and 15 × 30 cm and was at the least of the recipient-site defect size (Table 3).

**Table 3:** Surgical treatment–defect reconstruction with anterolateral thigh flap in 17 patients

	Number
Flap anatomy and type	
Fasciocutaneous	17
Perforators	
Musculocutaneous	11
Septocutaneous	6
Donor-site closure	
Direct closure	16
Skin graft	1
Length of pedicle (cm)	6–12 (mean 8)
Flap size (cm)	6 × 15 to 15 × 30
Flap thinned	2
Operative time (h)	5.8–9.5 (mean 7.3)
Flap ischemia time (min)	25–68 (mean 35)

In all cases except one, the donor-site defect was closed primarily. The patient with the largest defect extending to the scalp required a 15 × 30-cm flap. In this case, the size of the donor-site defect was too large for primary closure. The full-thickness skin graft from the inguinal region was used to cover this defect. In two cases, the flaps required subcutaneous fat thinning. No complication of thinning such as skin necrosis or bleeding from the under surface of the flap was observed.

Various recipient-site vessels were used for microvascular anastomoses. The following vessels were used on the resipient site: nine facial, five occipital and three maxillary arteries were utilised. Recipient-site veins included eight internal jugular, seven facial, one retromandibular and one external jugular vein. All arterial pedicles were anastomosed in an end-to-end manner, utilizing 9,0 or 10,0 nylon monofilament Ethilon^®^ interrupted sutures. No arterial grafts were required. End-to-end venous anastomoses were performed in 15 cases and end-to-side to the internal jugular vein in 2 patients.

There was no need for vein grafts. Both donor veins of the pedicle were anastomosed in 14 cases and only one of them was anastomosed in 3 cases. The dominant vein was utilised due to the very small diameter of the other one in the pair. In 6 patients, venous anastomoses were sutured with 9,0 or 10,0 nylon monofilament Ethilon^®^ interrupted sutures and in 11 cases, Synovis-Coupler® devices in sizes from 1.5 to 4 mm were used. All the flap transfers were performed successfully.

The duration of surgery from induction of anaesthesia to transfer to recovery room ranged between 5.8 and 9.5 h (mean 7.3 h). The mean flap ischemia time was 35 min (ranging from 25 to 68 min) (Table 3).

Four patients experienced postoperative complications. Two patients in the age over 60 years with a history of cardiac angina developed myocardial infarction one in 6 h and the other in 5 days after the surgery. Both recovered after endovascular treatment. In two patients, haematoma developed in the recipient operation site. The first one occurred 2 h after the successful management of the early postoperative myocardial infarction. The second patient developed a haematoma 10 h after the operation. Haematoma evacuation successfully salvaged the transplanted flaps in both patients. One patient experienced a partial flap necrosis that occurred 3 weeks after surgery. It was primarily managed by excision and secondary healing, followed by the full-thickness skin graft. There were no instances of cerebrospinal fluid leak, major vessel injury or damage to non-facial cranial nerves.

No impairment in range of motion and muscle strength of the donor leg occurred. The mean length of hospital stay was 10 days. It ranged between 7 and 32 days.

All patients left the hospital with a good volume filling of their defects and were generally satisfied with the cosmetic outcome.

## Discussion

In the treatment of lateral skull base malignant tumours, involving the temporal bone, the type and extent of operation is related to the tumour staging. In general, lesions confined to the external auditory canal T1 are treated with a local, limited resection. More advanced malignancies, T3 and T4, require en bloc removal by a lateral temporal bone resection, subtotal petrosectomy or total petrosectomy [1, 2, 5].

Small and, sometimes, medium-sized postoperative defects can be covered with local or regional flaps. Extensive resection of the temporal bone and its surroundings produces a massive defect of tissue disturbing the function and spoiling the cosmetic effect. Properly selected and executed reconstructions provide anatomical repair of the defects after extensive surgery of the lateral skull base. Healing of such large defects is not possible without immediate reconstruction using free tissue flaps [17, 18, 20, 21]. This become viable as a result of growing experience and refinements in surgical techniques, challenged by the complex three-dimensional anatomy, the relatively large bulk of the dead space and required pliability of the transplant. Locally, there is not enough tissue to perform adequate reconstruction. Therefore, transplants from distant sites became a viable solution.

Primarily, the rectus abdominis free flap and radial forearm free flap were used. The rectus flap is often too large with a short pedicle, requires debulking, and the donor site is prone to abdominal wall herniation. Despite these limitations, some centres favour this flap for lateral temporal bone defects [22, 23]. The radial forearm free flap presents a favourable characteristic for vessel geometry and is renowned for reliability. It is criticised for inadequate volume of tissue to seal a large three-dimensional defect. Another limitation is a risk of hand ischemia resulting from radial artery sacrifice, even though it is very rare. There are advocates claiming this flap to be reliable even in large lateral skull base defects when used in a double-layer fashion [14]. According to a recent paper of Thompson et al., radial forearm free flaps tended to have lower wound complication rates when compared with other techniques [15]. The last decades’ publications report the ALT flap to be a good option in the skull base and the scalp reconstruction; however, the reported experience in the repair of the lateral temporal bone defects is based on very small series [14–17, 23–28]. The ALT flap, described by Song et al., in year 1984, gained popularity [29]. It was claimed to be reliable, providing a large skin area, and harvested as either a fascio-cutaneous or a muscle-cutaneous flap. The thickness and volume of this flap are easily adjusted to match the defect. ALT flap can be harvested without changing intraoperative patient positioning allowing a single-staged surgery by the donor-site team and recipient-site team [24].

In our practice, the advantages of the ALT flap were successfully utilised to reconstruct the massive defect of tissue after subtotal petrosectomy in all patients. It was always possible to obtain a long pedicle with large vessels, giving a wide choice of recipient vessels. None of the patients needed a venous graft to connect the vessels. Also, the amount of available skin was adequate. Only in two of our cases, it was necessary to modify and reduce the flap volume. The favourable vascular anatomy of the flap permitted the thinning of the transplant without any complications.

One of the described limitations of the ALT flap is the skin colour that often does not match the colour of the recipient site. Therefore, the cosmetic effect is not always satisfactory; however, our patients accepted this disadvantage.

The overall complication rate of a variety of the free flaps used in reconstruction in head and neck surgery is 10–32% and free flap failure occurs in 2–9%. Complete flap loss is infrequent and is reported to lie below 5% of cases. Perioperative mortality rate is around 0.3% [26, 30–32].

The incidence of the complications of the lateral skull base tumour extensive resection followed by reconstruction specifically with ALT flap reported in the recent literature presents inconsistent opinions. The published series are based on small numbers of patients from 2 to 18 [14–16, 19, 23, 25–28]. Lin et al. used ALT flap in seven patients for reconstruction of lateral skull defect. At the recipient site, they reported infection in three patients, haematoma in one and additionally, two patients required revision for unsightly bulkiness. Complications at donor site occurred in three cases (two seromas and one haematoma) [14]. Thompson et al., in 11 cases out of 14, treated with ALT flap registered wound complications: infections, seromas and dehiscence [15]. Oranges et al. used the ALT flap for lateral skull base in ten patients. Complications occurred at the recipient site in six patients: infections in two, haematoma in two and dehiscence in two cases. They reported the donor-site complications in four patients, mainly due to dehiscence [16].

Our experience with reconstruction of extensive lateral skull base defects utilising the ALT flap did not confirm the high risk of complications neither at the recipient site nor at the donor site. None of the 17 patients experienced complication at the donor site. At the recipient site, complications were recorded in two patients who developed a local haematoma and one patient experienced a partial flap necrosis that occurred 3 weeks after surgery.

The cerebrospinal fluid leak complicating subtotal petrosectomy is rare. It is reported to occur in 5–20% in cases requiring dura resection [21, 24]. In our series, dura was resected and reconstructed in two cases and no CSF leaks occurred.

Cardiovascular disease and advanced age proved to increase the risk of flap ischaemia, even in cases of successful microvascular reconstructions [33]. This can be attributed to the poor quality of vessels, deprived compensation of blood loss and capacity to withstand lengthy surgical procedures. The age of over 60 years is recognised to be an important risk factor in the management of the temporal bone defects after oncologic resection [16]. In the described series of 17 patients, 12 were over 70 years of age. Within the first week after surgery, two of them developed myocardial infarction. The first patient developed the condition in 6 h, and the second patient developed the same condition 5 days after the operation. Both had a history of cardiac angina. The first patient, during the treatment of postoperatively occurring myocardial infarction, developed a haematoma at the site of tumour removal. This might be attributed to anticoagulation therapy introduced during coronaroplasty Both patients experiencing postoperative myocardial infarction recovered and left the hospital in a good clinical condition.

The resection of lateral skull neoplasms in the presented series did not include facial nerve grafting or innervation in all patients. The patients were affected by multiple comorbidities, advanced age and tumour stage. The addition of a free flap, facial reinnervation and reanimation adds considerable operative time to an often lengthy extirpative procedure. As a result, the risk of postoperative complications may be increased in those patients with significant comorbidities [34, 35].

The ALT flap gains popularity in reconstructive surgery; however, its use in covering extensive defects in the lateral skull base is still limited. The number of publications and size of examined cohorts is small and inhomogeneous [14, 15, 23–28]. ALT flap proved to be effective, but recent publications report relatively high rate of complications. On the contrary, in the reported study, the complications were rare. The differences in the reported level of risk may be due to small groups of operated patients, variety of their clinical status and in surgical details.

The patients who are most likely to benefit form the ALT flap are those in whom soft tissue defect including skin is massive after extensive resection of lateral skull base tumours. This flap should be also considered in patients with comorbidities increasing the risk of donor-site complications.

The management of lateral skull base malignancies remains to be a challenge in head and neck surgery. Progress and accumulated experience with free flap reconstruction enable more radical tumour excision and better outcomes.

## Conclusions

The ALT free flap proved to be effective for covering large temporal skull base defects resulting from the radical removal of temporal bone malignancies. The surgical procedures are safe and their results are acceptable. The complications at the recipient and the donor sites are rare and manageable.
